# Large Field Screening for Resistance to Broomrape (*Orobanche crenata* Forsk.) in a Global Lentil Diversity Panel (GLDP) (*Lens culinaris* Medik.)

**DOI:** 10.3390/plants12102064

**Published:** 2023-05-22

**Authors:** Youness En-nahli, Kamal Hejjaoui, Rachid Mentag, Nour Eddine Es-safi, Moez Amri

**Affiliations:** 1 AgroBioSciences Program (AgBS), College of Sustainable Agriculture and Environmental Science (CSAES), University Mohammed VI Polytechnic (UM6P), Ben Guerir 43150, Morocco; 2 Materials Science Center (MSC), Mohammed V University in Rabat, Laboratoire de Physicochimie de Matériaux Organiques et Inorganiques (LPCMOI), Ecole Normale Supérieure, Rabat 10010, Morocco; 3 Biotechnology Research Unit, Regional Center of Agricultural Research of Rabat, National Institute of Agricultural Research (INRA), Avenue Ennasr, Rabat 10000, Morocco

**Keywords:** *Orobanche crenata*, *Lens culinaris*, screening, resistance, grain yield

## Abstract

Broomrape (*Orobanche crenata* Forsk.) is a serious problem causing important losses to lentil (*Lens culinaris* Medik.) production and productivity in Mediterranean countries. Despite intensive breeding activities, no resistance sources against *O. crenata* have been identified so far. In this study, a Global Lentil Diversity Panel (GLDP) of 1315 genotypes including local populations, landraces, accessions, improved lines and released varieties were evaluated for their resistance to *O. crenata* under highly infested field conditions at ICARDA Marchouch research station, Morocco. The trial was conducted according to an augmented design with repeated susceptible checks. The best-performing genotypes were selected based on the correlations between *Orobanche* infestation parameters and agronomic performance. Results showed significant variation (*p* < 0.005) among the studied genotypes and between the tested genotypes and checks for BY, D2F, D2M, PH, EODW and NEO. Out of the 1315 tested genotypes, only (1%) showed high to moderate resistance levels to *O. crenata*. Most of these genotypes are improved lines originating from different breeding programs. the PCA analysis clustered all the tested genotypes into four different groups. Good resistance levels were recorded for the genotypes ILL7723, ILL 7982, ILL 6912, ILL 6415, ILL 9850, ILL 605, ILL 7915, ILL 1861 and ILL 9888 showing a parasitism index and grain yield ranging from 1.69 to 5.99 and 10.97 to 60.19 g m^−2^, respectively. Person’s correlation showed significant negative correlations between agronomic traits and infestation parameters. Both the path and spatial analysis showed that the D2F, NEO, D2OE, SEV and parasitism index (PI) were the strongest driver traits that influenced the seed yield (SY).

## 1. Introduction

Lentil (*Lens culinaris* Medik.) is one of the major cool-season legume crops, bringing an important added value for sustainable agriculture for cereal-based systems due to its capacity to fix nitrogen and improve soil fertility [1,2]. It has been traditionally cultivated in many regions of the world including West and South Asia, Sub-Saharan Africa, North Africa, Southern Europe, the Americas and Oceania [3,4]. With more than 40,000 ha of cultivated area and of 30,670 tons total annual production, Morocco is the second-largest lentil producer in Africa and the most important producer in the Mediterranean region [5]. Climate change, with all the associated stresses such as the irregularity of precipitations and their distribution, an increasing temperature and the development of new, more aggressive and virulent diseases and pests, has resulted in an important decrease in lentil production and productivity. In the Mediterranean region, the fast spread of the parasitic weed *Orobanche crenata* remains one of the main constraints that causes severe yield losses [6]. Under highly infested conditions, attacks by *O. crenata* can completely destroy the crop and result in important economic losses for farmers [7,8].

Several control methods have been tested for broomrape management [6,9,10]. Hand weeding has shown to be expensive, time-consuming and impractical under high infestation conditions, but it has very limited success as most of the parasite damage on the host plant occurs before emergence [11]. Despite its effectiveness compared to other control methods, chemical control using herbicide application has a negative impact on the environment and human health and can not be adopted as a long-term control strategy [9,12]. Other methods, such as catch crop, bio-stimulants, suicidal germination, intercropping, sowing date and solarization have been recommended to control *Orobanche* and reduce the tremendous seed bank in the infested field [13,14,15,16]. However, all these methods have shown limited success in broomrape management and control, especially among poorly resourced farmers [6,8,17]. Breeding for resistance remains the most successful, effective and environmentally friendly control method for *Orobanche* management [6,12,14]. The selection and development of resistant varieties is considered to be a simple technology that is easy to adopt by farmers. For some legume crops such as faba bean (*Vicia faba* L.), many resistant varieties have been developed and released [18,19]. These varieties have been largely adopted by farmers and have significantly contributed to improving production and productivity under infested conditions [11,12,13,18,19,20]. Unfortunately, to date, no lentil varieties with resistance to broomrape have been developed and released because of the limited resistance sources and the low heritability of associated resistance traits [12]. A very limited number of studies have tried to explore genetic diversity in the lens genus for potential resistance sources to parasitic weeds (in particular *O. crenata*) [21,22]. In a recent study, En-nahli et al. (2021) [7], reported some potential resistance sources to *O. crenata* in a small cultivated lentil collection and explored some potential biochemical resistance mechanisms.

The main objectives of this study are to (i) explore the genetic diversity and evaluate the response of a Global Lentil Diversity Panel (GLDP) to *O. crenata* parasitism under field conditions, and (ii) identify potential sources that can be used in future breeding programs.

## 2. Results

### 2.1. Variance, Descriptive Statistic and Spacial Analysis

Results showed high genotypic variation within the lentil germplasm collection in response to *O. crenata* parasitism. Significant differences (*p* ≤ 0.05) were observed between the tested genotypes for BY, D2F, D2M, EODW, NEO, and PH. No significant differences were recorded for DOE, INC, SEV and SY.

Significant differences (*p* ≤ 0.05) were also recorded between all tested genotypes and the check for the BY, D2F, D2M, EODW, NEO, and PH. Substantial variations among the checks for DOE, EODW, INC, NEO, SEV and SY were also observed. Except for *Orobanche* incidence (INC), significant variability was found among genotypes in the different blocks for all other parameters (Table 1).

Results showed a high genotypic variation among all studied genotypes based on agro-morphological traits (Table 2). The D2F ranged from 61 to 111 days with a mean value of 85 days, and the D2M ranged from 115 to 145 with an average of 127 days. The mean values of BY and SY were 211.45 and 7.05 g/m^2^, respectively. The biological yield (BY) varied from 0 to 1797.94 g/m^2^ and the SY varied from 0 to 108.13 g/m^2^.

High genotypic variation was also observed for the infestation parameters. The number of days to *Orobanche* emergence (DOE) ranged from 70 to 109 days, with an average of 88 days. The maximum DOE was observed for the genotype ILL 69,498 against a minimum observed for the genotype Bakria. The parasitism index (PI) calculated using the incidence (INC) and severity (SEV) varied from 0 ta 9 with a mean value of 7 recorded for the whole tested collection. The number of emerged *Orobanche* per plant (NEO) ranged from 0 to 168 with an average of 88 recorded for the whole collection, and the emerged *Orobanche* dry weight (EODW) per plant ranged from 0 to 114.25 g with an average of 1.13 g.

**Figure 1:**
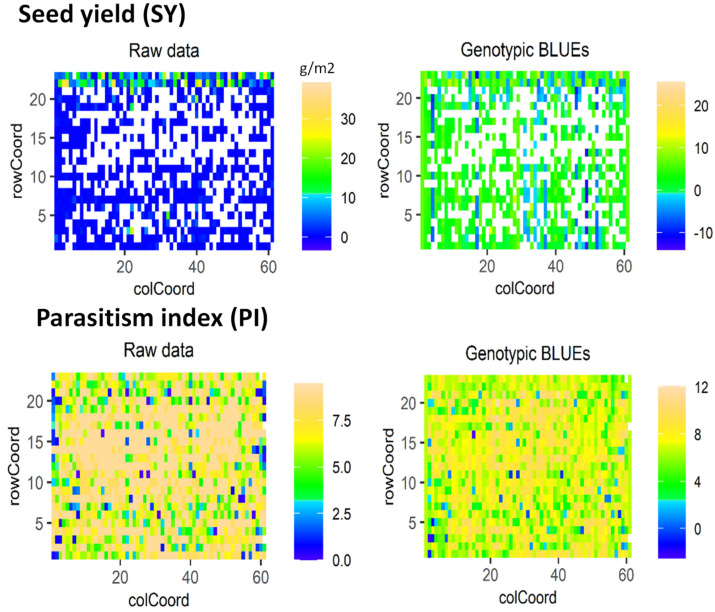
Spatial analysis using row and column coordinates for seed yield (SY) and parasitism index (PI) showing the raw data, best linear unbiased estimates (BLUEs) and genotypes distribution frequency.

Spatial analysis using row and column coordinates adopted in the experimental design allowed us to identify spatial field distribution patterns for PI and SY (Figure 1). The tested genotypes showed a normal distribution frequency for SY. Almost 99% of the tested genotypes showed SY less than 10 g/m^2^ with a complete crop loss observed for most of the genotypes. High parasitism impact was observed in the different rows and columns of the experimental field. About 90% of the tested genotypes showed a PI higher than 5. Out of the total 1315 tested genotypes, only around 12 genotypes (1%) showed moderate to high resistance levels.

### 2.2. Relationship between Agronomic and Infestation Parameter

**Figure 2:**
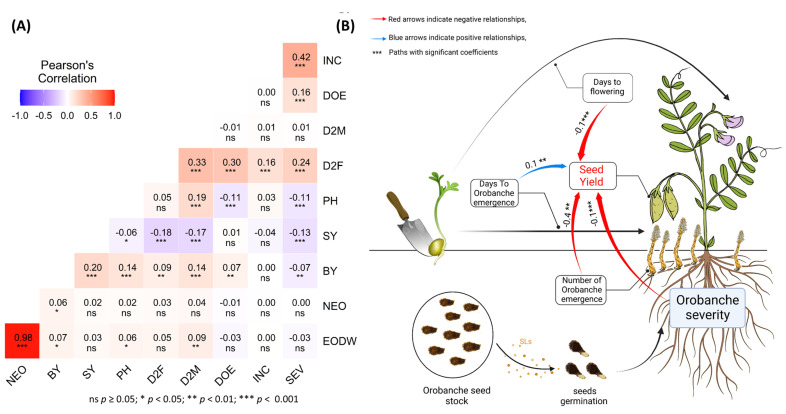
(**A**) Correlation analysis among morphological parameters of lentil in response to *O. crenata* infection under field conditions during the cropping season 2018–2019. (**B**) The path analysis numbers adjacent to the arrows indicate the relationship effect size and the stars are associated with the bootstrap *p*-value. Blue and red arrows indicate positive and negative relationships, respectively; only significant paths are presented. D2F: Days to flowering; DOE: Days to *Orobanche* emergence; EON: Emerged *Orobanche* number; EODW: Emerged *Orobanche* dry weight; BY: Biological yield; SY: Seed yield. *** Correlation is significant at the 0.001 level; ** Correlation is significant at the 0.01 level; * Correlation is significant at the 0.05 level.

The pairwise correlation matrix (Figure 2A) revealed that *O. crenata* severity (SEV) was negatively and significantly correlated with SY (r = −0.13 ***) and BY (r = −0.07 ***). Negative correlations were also observed between SY and D2F (r = −0.18 ***). However, D2F showed a positive correlation with DOE (r = 0.33 ***). *Orobanche* incidence (INC) was positively and significantly correlated with SEV (r = 0.42 ***). A negative significant correlation (r = −0.11 **) was observed between DOE and PH. However, DOE showed positive and significant correlations with BY (r = 0.07 **) and D2F (r = 0.30 ***). D2M showed positive correlations with EODW, BY, PH and D2F with a correlation coefficient of (r = 0.09 **), (r = 0.14 ***), (r = 0.19 ***) and (r = 0.33 ***), respectively. NEO revealed a highly positive correlation with EODW (r = 0.96 ***) and a positive correlation with BY (r = 0.06 *).

To provide detailed and reliable insights into the complex causal relationships underlining lentil seed yield production with agronomic and infestation parameters, a structural equation modeling (path analysis) was performed (Figure 2B). The path coefficients were obtained using the path analyses and provided a clear view and interpretation of the cause–effect relationships between the various agronomic traits and infestation parameters and their relative impact on lentil seed yield. The results showed that the strongest driving parameters affecting lentil SY were SEV (r = −0.1 ***) and NEO (r = −0.4 **). However, the delay of *Orobanche* emergence expressed by the number of days to Orobanche emergence (DOE) was reflected by a positive effect on lentil SY (r = 0.1 **).

### 2.3. Multivariate Analysis

**Figure 3:**
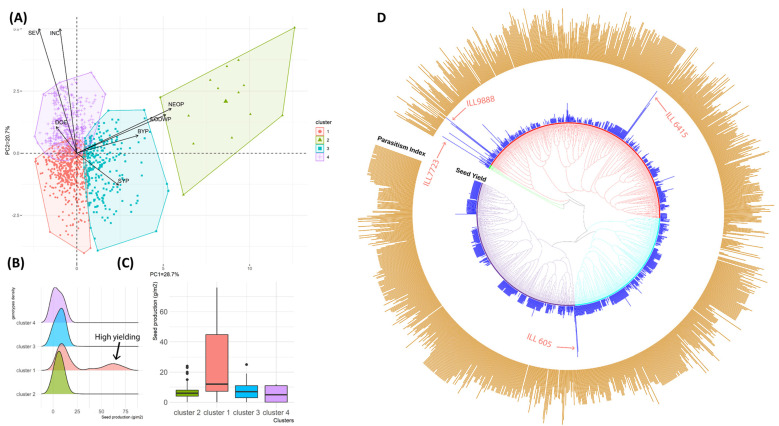
(**A**) Biplot of the first two dimensions of the principal component analysis (PCA) for the 1315 genotypes based on their agronomic and pheno-physiological performance under *O. crenata* infection under open field conditions. (**B**) Density plot showing the distribution of genotypes for each cluster based on seed yield (SY) (**C**) Box plots showing the SY comparison between the different clusters (**D**) Cladogram showing relationships between PI and SY recorded for the total of 1315 lentil germplasms.

**Figure 4:**
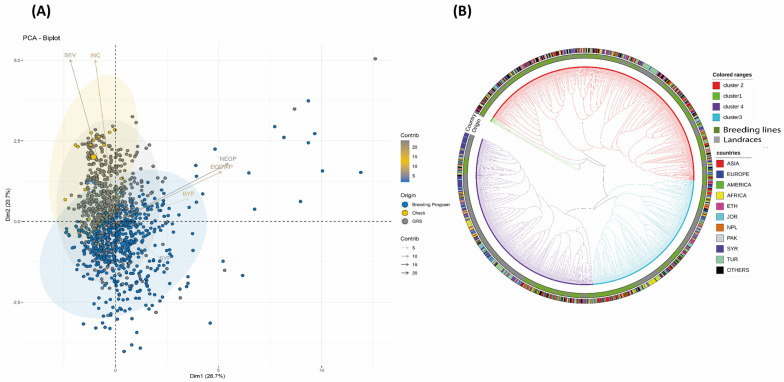
**(A**): Biplot of the first two dimensions of the principal component analysis (PCA) for the 1315 genotypes based on their morphological traits under *O. crenata* infection in field conditions clustered according to their origins. (**B**) Cladogram showing relationships between 1315 lentil germplasm according to the agronomic performance and their origins.

The agronomic performance of different tested genotypes and their correlations with *Orobanche* infestation traits were confirmed at a higher dimension using principal component analysis (PCA) (Figure 3A). The two first principal components of the PCA (PC1 and PC2) explained 49.4% of the total variation. The PC1 (28.7%) was positively correlated with BY, SY, NEO and EODW and the PC2 (20.7%) was negatively correlated with SY but positively correlated with the other traits (BY, EON, EODW, DOE, INC and SEV). The PCA grouped the total 1315 tested genotypes into four different groups (Figure 3B,C). The first cluster with 520 genotypes mainly originated from breeding lines. This Cluster grouped most of the genotypes that showed good to moderate resistance to *O. crenata*. Cluster 2 was more correlated with NEO and EODW and grouped all the genotypes with high infestation levels. The genotypes in this cluster basically originated from breeding lines. Cluster 3 was more correlated with SY and BY and grouped genotypes that originated from both breeding lines and landraces/accessions. Cluster 4 was more correlated with SEV and INC and grouped genotypes all with the susceptible checks that showed high susceptibility to *O. crenata* parasitism. Most of the genebank collection including local populations, landraces and accessions were grouped in this Cluster 4 (Figure 4A,B). Specifically, distribution and box plots (Figure 3B,C) showed that Cluster 1 contains the majority of high-yielding genotypes (≥50 g/m^2^) compared to the other clusters.

Both SY and PI were plotted together as bar plots in the cladogram (Figure 3D) that was used to identify genotypes with good resistance to *O. crenata* expressing relatively high SY and low PI. Out of the total 1315 tested genotypes, only around 1% (12 genotypes) showed a good resistance level to *O. crenata*. Among these genotypes, ILL 9888, ILL 6415, ILL 7723, ILL 605, ILL 6912, ILL 8198, ILL 7915, LIRL21-187and ILL 7982 expressed the highest resistance levels with SY ranging from 12.28 to 60.19 g/m^2^ and PI varying from 1.69 to 5.55. Most of the genotypes that showed the highest level of resistance with low PI and high SY originated from breeding programs such as ILL 6912, ILL 605, ILL 7915 and ILL 9888 the other genotypes included landraces and accessions received from genebanks such as ILL 6415, ILL 9850 and ILL 1861 (Figure 4A,B).

## 3. Discussion

In this study, we evaluated the performance of a large lentil germplasm collection under high *O. crenata*-infested field conditions. High genotypic variation was observed within the tested germplasm collection. *O. crenata* parasitism significantly affected lentil growth and productivity. A clear negative correlation was recorded between SY and PI, which can explain the low SY level observed for most of the genotypes with high PI. Several previous studies have reported a similar negative impact of broomrape on lentil [3,4,6,21], fava bean (*Vicia faba* L.) [11,12,22,23,24,25], chickpea (*Cicer arietinum* L.) [26,27] and grass pea (*Lathyrus* spp.) [28].

The number of emerged *Orobanche* shoots (NEO) was strongly correlated with emerged *Orobanche* dry weight (EODW) (r = 0.98 ***). This shows that, regardless of the infection level, the mean size of the *O. crenata* shoots infecting a single lentil plant is essentially similar to the range of infection. A similar strong correlation between NEO and EODW was observed in fava bean infected by *O. crenata* [29,30]. The number of emerged *Orobanche* shoots (NEO) was the main driver of the negative *O. crenata* parasitism impact on SY (r = −0.4 **) (Figure 2B). The attachment of many *Orobanche* tubercles/shoots to the host root system results in increasing the parasitism impact and severity (SEV) through increased uptake of water and nutrients from the host [24]. The limited number of emerged broomrape shoots was previously used as an indicator of resistance to broomrape [11,12,30]. This could be considered to be a criterion for selection only under low *Orobanche*-infested conditions (Amri M., personal communication). Under heavy infestation, the high competition for water and nutrients between a large number of underground *Orobanche* tubercles, especially for susceptible genotypes, could result in low emerged or even non-emerged *Orbanche* shoots (Amri M., personal communication). In our study, the low infestation level expressed by low PI was combined with the SY to select the best genotypes carrying resistance to *O. crenata*.

The BY was also significantly affected by *O. crenata* parasitism with a negative correlation observed especially with the SEV of infection. Such reduction in the host biomass production might be related to the metabolic competition between the host biomass production and defense [17]. This correlation between host BY and *Orobanche* severity (SEV) has been previously reported also in fava bean under *O. crenata* parasitism [29]. This interaction also explains the negative and significant explanatory impact (r = −0.1 ***) observed between SEV and SY. A recent study showed that *O. crenata* reduced the biomass of susceptible and resistant fava bean and lentil cultivars [31]. The same authors reported that the dry matter production and allocation under *O. crenata* parasitism is mainly attributed to a source-sink interaction between the host and its parasite.

Early flowering seems to play a major role in host–parasite interaction. Our results showed a high correlation between the *Orobanche* infection parameters (NEO, DOE, EODW, SEV and INC) and the number of days to flowering (D2F) and indirectly the SY. Early flowering is usually associated with early metabolomic adjustments and early release of *Orobanche* seed germination stimulants in the root rhizosphere of the host plant. This results in early germination and the attachment of parasite tubercles to the host root system. This will be reflected later by an increased negative parasitism impact on the host plant [7,12]. By contrast, other studies [22,32] have reported that early flowering and early maturing fava bean genotypes had the ability to escape broomrape damage due to early pod setting and filling. This strategy has already been described in faba bean and lentil and could be explained by a source-sink competition between the parasite and pods [22,33].

Breeding for resistance to broomrape in legume crops has accelerated in recent decades. Since then, many sources of resistance have been identified and several resistant varieties have been released in fava bean [11,18,19,34,35,36], chickpea [26] and grass pea [28]. In lentil, some authors recently reported moderate resistance in the cultivated variety Bakria (ILL 4605) [4]. In our study, and out of the total of 1315 tested, the genotypes ILL 9888, ILL 6415, ILL 7723, ILL 605, ILL 6912, ILL 8198, ILL 7915, LIRL21-187 and ILL 7982 showed high resistance to *O. crenata* (Figure 3D). Such resistance was expressed by the limited development of the parasite, which was reflected by low *Orobanche* infestation and good capacity to produce grains (SY) under highly infested conditions.

In general, the lack of complete resistance, the additive effect and polygenetic control of the resistance, and the low heritability of the genes/QTLs associated with resistance to broomrapes remains the most challenging problem in breeding for resistance to *Orobanche* and *Phelipanche* in legume crops. In this study, the identified resistant genotypes could be used as potential resistance sources in future breeding activities for *Orobanche* resistance in lentil. These resistance sources need further exploration of different involved molecular and biochemical resistance mechanisms, the associated genes/QTLs, and their potential introgression into well-adapted high-yield varieties.

## 4. Material and Methods

### 4.1. Plant Material and Field Trial

**Figure 5:**
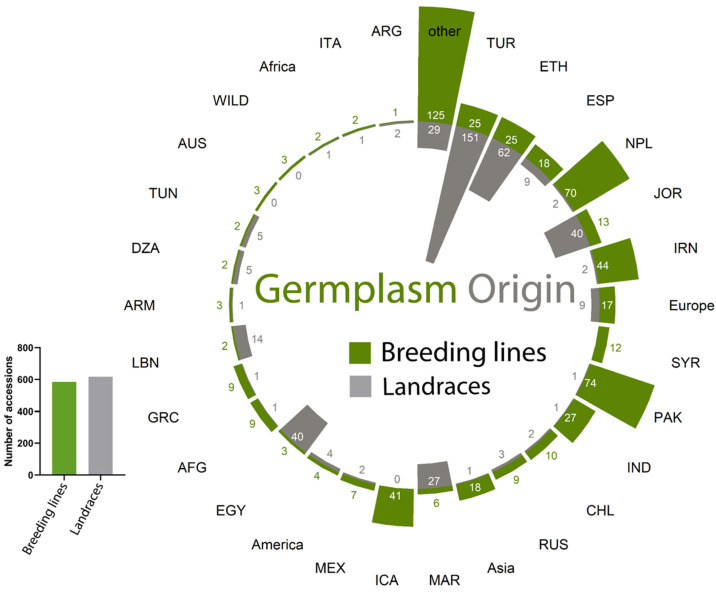
Lentil genotypes origin used to identify the resistance levels under field conditions during the cropping season 2018/2019.

A Global Lentil Diversity Panel of 1315 genotypes including improved lines, accessions, landraces and released varieties was subjected to large field screening and evaluation for resistance to *O. crenata* (Figure 5). The germplasm collection was provided by the International Center of Agricultural Research in dry areas genebank—ICARDA, Rabat. The trial was conducted during the cropping seasons in 2018/2019 in a highly *O. crenata*-infested plot at ICARDA Marchouch research station, Morocco. Different genotypes were planted at the end of November according to an augmented design with repetitive checks. For each genotype, 30 seeds were planted in 1 m rows with 30 cm inter-row spacing. The local cultivar Bakria (ILL 4605), which is reported to be moderately resistant to *O. crenata* [31], was used as a repeated check. No herbicides or fertilizers were applied throughout the crop cycle. Hand weeding was undertaken where necessary.

The following pheno-physiological and agronomic parameters were recorded before and at crop maturity. Number of days to flowering (D2F), days to *Orobanche* emergence (D2OE), *Orobanche* incidence (OIN), *Orobanche* severity (OSV), Emerged *Orobanche* number per plant (EON), Emerged *Orobanche* dry weight per plant (EODW), biological yield g m^−2^ (BY) and seed yield g m^−2^ (SY) and parasitism index (*PI*). The PI was calculated according to the following formula:

(1) $$PI=\left(OIN\times OSV\right)/100$$

OIN: Percentage of lentil plants showing at least one emerged shoot of *Orobanche* per row. OSV: level of damage (1–9 scale) caused by the parasite on lentil growth and seed production [37].

### 4.2. Exploratory Data Analyses

Descriptive statistics, including the number of observations (*n*), mean, standard deviation (sd), median, absolute deviation (mad), minimum (min), maximum (max), and standard error (se) as well as plotting frequency distributions were performed using the psych package [38]. Pearson’s rank correlation was calculated in R using the function “ggcorr” included in the package corrplot [39]. For each trait, analysis of variance was retrieved using the “augmentedRCBD” R package to evaluate the relative contribution (RC) of genotype and experiment. The model assumed the genotype and replicate effects as fixed and the block effect as random. The best linear unbiased estimates (BLUEs) for each genotype were calculated and used for further statistical analysis. Spatial analysis was performed using “statgenSTA” and “statgenGxE” packages in Rstudio software to improve the precision of the estimated genotype effects and contrasts and to examine the heterogeneity patterns of field infestation. Principal component analysis (PCA) and hierarchical clustering (HCA) was carried out using “factoextra” and “FactoMineR” packages implemented in Rstudio. PCA and HCA are used to identify patterns in data and show the dissimilarities and similarities underlying the dataset. PCA was also used to indicate the relationships between groups of variables in the dataset and show relationships existing between the different studied traits. Cluster analysis used for the grouping of each object into clusters was performed using Ward’s method based on Euclidean distance over a complete linkage dissimilarity matrix.

## 5. Conclusions

In recent decades, the fast spread of *O. crenata* in farmers’ fields in the Mediterranean region has affected lentil production in Morocco as well as many other African and Mediterranean countries. In the absence of effective control methods, the identification and selection of resistant varieties remains the cornerstone of any potential integrated control strategy. In this study, through a large field screening, only 1% of the total 1315 tested accessions showed good resistance to *O. crenata*. This list includes the genotypes ILL 9888, ILL 6415, ILL 7723, ILL 605, ILL 6912, ILL 8198, ILL 7915, LIRL21-187 and ILL 7982. Most of these genotypes showed a good capacity in limiting the development of the parasite and producing seeds under highly infested field conditions. Such sources of resistance could be very useful in future breeding programs and the development of resistant varieties, which could open new insight into limiting the spread of these parasitic weeds and alleviating their damage to legume crops. However, a better understanding is required of the chemical, molecular, and genetic factors underlying the resistance in these selected genotypes during the host–parasite interaction; in particular, the role of strigolactones and various *haustorial* initiation factors in the control of pre-attachment interactions is needed.

## Figures and Tables

**Table 1:** Analysis of variance of morphological traits of lentil genotypes under *O. crenata* infestation conditions.

Source	BY	D2F	D2M	DOE	EODW	INC	NEO	PH	PI	SEV	SY
Block	24,993.2 **	557.4 **	32.7 **	324.9 **	18.9 **	978.7 ns	30.0 **	125.6 **	20.1 **	15.8 **	2518.1 **
Genotype	13,144.8 **	103.4 **	21.1 **	21.5 ns	10.6 **	399.4 ns	21.8 **	24.82 **	4.4 ns	1.9 ns	74.6 ns
Check	29,319.6 **	7.9 ns	4.9 ns	185 **	5.7 **	6667.0 **	3.2 **	11.8 ns	54.5 **	18.2 **	1300.1 **
Gen vs. Check	13,107.8 **	103.6 **	21.2 **	21.2 ns	10.6 **	385.0 ns	21.8 **	24.8 **	4.3 ns	1.9 ns	71.8 ns
Residuals	6583.9	5.2	4.8	19.7	0.4	596.4	0.3	9.5	6.2	2.9	167.9

D2F: Days to flowering, DOE: Day to *Orobanche* emergence, PI: Parasitism index, EON: Emerged *Orobanche* number, EODW: Emerged *Orobanche* dry weight, BY: Biological yield, SY: Seed yield, ** significant at the 0.01 level; ns not significant.

**Table 2:** Range, mean and standard error (SE) recorded for different agronomic and *O. crenata* infestation parameters.

Trait	Count	Mean	SE	Min	Max
D2F	1315	85	0.3	60	111
D2M	1315	127	0.1	115	145
DOE	1315	88	0.2	70	109
PH	1315	28.8	0.2	14	46.3
NEO	1315	1.2	0.1	0	168
EODW	1315	1.13	0.1	0	114.3
INC	1315	91.4	0.7	0	138.1
SEV	1315	8	0.1	2	9
PI	1315	7	0.1	0	9
BY	1315	211.5	3.3	0	1797.9
SY	1315	7.1	0.2	0	108.1

D2F: Days to flowering, DOE: Day to *Orobanche* emergence, PI: Parasitism index, EON: Emerged *Orobanche* number, EODW: Emerged *Orobanche* dry weight, BY: Biological yield, SY: Seed yield.

## Data Availability

The data presented in this study are available on request from the corresponding author.
